# The increase in activating EGFR mutation in plasma is an early biomarker to monitor response to osimertinib: a case report

**DOI:** 10.1186/s12885-019-5604-6

**Published:** 2019-04-30

**Authors:** Marzia Del Re, Eleonora Rofi, Carla Cappelli, Gianfranco Puppo, Stefania Crucitta, Simona Valeggi, Antonio Chella, Romano Danesi, Iacopo Petrini

**Affiliations:** 1 Unit of Clinical Pharmacology and Pharmacogenetics, Department of Clinical and Experimental Medicine, University of Pisa, 55, Via Roma, 56126 Pisa, Italy; 2 Unit of Diagnostic and Interventional Radiology, Department of Translational Research and New Technologies in Medicine and Surgery, University Hospital of Pisa, Pisa, Italy; 3 Unit of Respiratory Medicine, Department of Critical Area and Surgical, Medical and Molecular Pathology, University Hospital of Pisa, Pisa, Italy

**Keywords:** Circulating tumor DNA, NSCLC, EGFR mutations, Treatment monitoring, EGFR-TKIs, Digital droplet PCR, NGS

## Abstract

**Background:**

Systemic treatment of advanced non-small cell lung cancer (NSCLC) has changed dramatically since the introduction of targeted therapies. The analysis of circulating tumor DNA (ctDNA) is a valuable approach to monitor the clonal evolution of tumors during treatment with EGFR-tyrosine kinase inhibitors (TKIs) and to detect resistance mutations.

**Case presentation:**

A NSCLC patient with exon 19 deletion (ex19del) of EGFR was treated with osimertinib after multiple lines of treatment and obtained a partial response that lasted over 26 months. Blood was collected at each visit and ctDNA was extracted to monitor ex19del by digital droplet PCR. Within a few weeks from the beginning of osimertinib, ex19del disappeared from plasma but appeared again and steadily increased a few months later anticipating tumor progression. Interestingly, the change in ex19del was much more pronounced than other mutations, since T790M appeared 3 months after the increase of ex19del, and C797S was detectable a few weeks before clinical disease progression. Then the patient received cytotoxic chemotherapy, which was associated with a decrease in ex19del and disappearance of T790M and C797S; however, at disease progression, all EGFR mutations increased again in plasma together with MET amplification which was detected by NGS.

**Conclusions:**

The measurement of ex19del changes in ctDNA is a simple and sensitive approach to monitor clinical outcome to osimertinib and, potentially, to other therapeutic interventions.

## Background

The presence of activating EGFR mutations, mainly ex19del, strongly predicts response to EGFR-TKIs; however, in 50–60% of these patients, resistance is acquired through the development of T790M, a second missense mutation of EGFR, which is indeed targeted by osimertinib [1]. Some patients retain EGFR oncogene addiction even after progression to osimertinib, as they may develop the C797S resistance mutation [2, 3].

The analysis of circulating tumor DNA (ctDNA) is a valuable approach to monitor the clonal evolution of tumors during treatment and to detect mutations capable of inducing resistance to EGFR-TKIs [4]. Even if the analysis of tumor tissue is required to select the appropriate treatment, it is indeed associated with several limitations, including invasiveness, inability to comprehensively capture tumor heterogeneity, and tissue availability for mutational testing. For these reasons, the analysis of EGFR mutations in ctDNA has recently emerged as a reliable, non-invasive alternative approach, showing high concordance with tissue molecular profile, with good sensitivity (> 65%) and high specificity (> 88%) [5].

Here, we report a case of ctDNA monitoring during osimertinib treatment and after disease progression, which provides evidence of the reliability of time-dependent changes in EGFR activating mutation to predict response to treatment.

## Case presentation

**Fig. 1:**
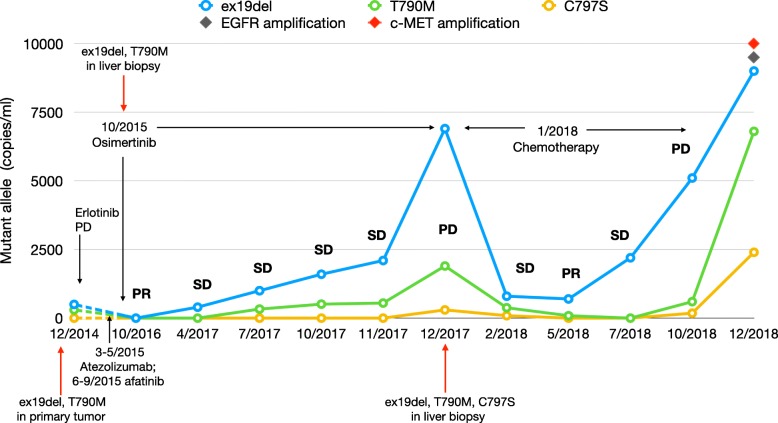
Changes of EGFR mutations (copies/ml) in ctDNA of patient during treatments. PR: partial response; SD: stable disease; PD progressive disease. The numbers before the year indicate the months

In October 2012, a 46-year-old woman was referred to our center for the presence of a large mass (50 × 70 mm) in the superior lobe of the left lung with homolateral pleural effusion. The patient was never smoker, without family history of cancer and without comorbidity. The cytological diagnosis was made using a CT-guided fine needle aspiration of the primary tumor and revealed an adenocarcinoma of the lung (TTF1+, CK7+) with the EGFR ex19del mutation. A PET-CT demonstrated the presence of liver and bone metastases and a nodule in the right breast, confirmed as a metastasis by fine needle aspiration. The patient received zoledronic acid 4 mg every 28 days and gefitinib 250 mg daily since November 2012 obtaining a partial response (PR). In August 2013, a disease progression (PD) was documented, with an increase in size of the primary tumor and size and number of liver metastases. A brain MRI revealed the presence of two cortical nodules, which were treated with stereotactic radiotherapy. The patient was enrolled in the IMPRESS trial and received 6 cycles of cisplatin and pemetrexed plus gefitinib obtaining again a PR that lasted until June 2014. Thereafter, a new lung metastasis appeared in the superior lobe of the left lung and the mammary nodule increased in dimensions. From June 2014 to December 2014 the patient received erlotinib 150 mg daily obtaining an initial stabilization of the disease (SD); however, within 6 months, she experienced again a PD with the increase of the mammary nodule and the appearance of a new bone metastasis in the sacrum. In December 2014, EGFR ex19del and T790M mutations were detectable in a new needle biopsy of the primary tumor; only at this time a digital PCR-based method was available for the analysis of circulating tumor DNA (ctDNA). Briefly, the method was optimized in order to recover a suitable amount of ctDNA for molecular analysis from 3 ml of plasma using the QIAmp Circulating Nucleic Acid Kit (Qiagen®, Valencia, CA). ctDNA was examined using the Prime PCR Probe Assay on a QX100™ Droplet Digital™ PCR System (BioRad®, Hercules, CA) for EGFR mutations (ex19del, T790M, and C797S) [6]. The ctDNA sample was considered as EGFR mutant when at least one droplet was above the fluorescence intensity threshold of 3000 and results were reported as copies/ml. The first plasma specimen was obtained in December 2014 and confirmed the presence of ex19del and T790M mutations (480 and 260 copies/ml, respectively; Fig. 1). The patient was treated with atezolizumab from March to May 2015 and received stereotactic radiotherapy on the lung primary tumor and on metastases of the left superior lobe, breast and bone (sacrum and D10). Due to PD, the patient was given afatinib 40 mg daily from June 2015 to September 2015, but she experienced a further PD with an increase in size of the primary tumor and lung nodules, the appearance of new multiple bilateral lung metastases, lymphangitic infiltration and liver metastases. At this time, the presence of ex19del and T790 M was again demonstrated in a biopsy of a liver metastasis. Since osimertinib was available, the patient was enrolled in the ASTRIS trial and given 80 mg daily starting from October 2015 with an immediate clinical benefit. At the first evaluation a PR was documented with disappearance of most of the lung nodules and lymphangitic infiltration, reduction of the primary tumor and of liver metastases as well. A disappearance of ex19del or T790M was demonstrated in ctDNA in a blood sample obtained in October 2016. However, in April 2017, ex19del appeared again (400 copies/ml) and in July 2017 it increased to 1000 copies/ml, while T790M was also detectable (330 copies/ml, Fig. 1). Then, ex19del and T790 M continued to increase to, respectively, 1600 and 510 copies/ml in October 2017, 2100 and 550 copies/ml in November 2017, and 6900 and 1900 copies/ml in December 2017.

A radiological progression was demonstrated with increase in size and number of liver metastases in December 2017. The patient underwent a new liver biopsy that confirmed the PD and the presence of ex19del and T790M, whereas the ctDNA showed also the appearance of C797S mutation (170 copies/ml), in addition to ex19del and T790M. The patient started chemotherapy with carboplatin and pemetrexed and in February 2018 she obtained an SD associated with a drop of ex19del (800 copies/ml), T790 M (380 copies/ml), and C797S (90 copies/ml) and then a PR in May 2018, with disappearance of C797S and reduction of ex19del (760 copies/ml) and T790M (90 copies/ml). In July 2018, however, ex19del strongly increased to 2200 copies/ml, even though T790M and C797S were undetectable. Finally, in October 2018, when a PD was documented, ex19del increased to 5100 copies/ml, while T790M and C797S appeared again in plasma with 600 and 180 copies/ml, respectively (Fig. 1). At this time a NextSeq 550 NGS platform (Illumina®, San Diego, CA) was available to analyse ctDNA by the AVENIO ctDNA Expanded Kit (Roche®, Pleasanton, CA). A plasma sample collected in December 2018 and analysed by NGS confirmed the presence of the ex19del, T790M and C797S and found, in addition, EGFR and c-MET amplifications, which were not present in tissue in the last re-biopsy of December 2017.

## Discussion and conclusions

Molecular mechanisms underlying the acquired resistance to first- and second-generation EGFR-TKIs have been characterised and include MET amplification, T790M, BRAF and PIK3CA mutations, AXL overexpression and transformation to SCLC or squamous cell carcinoma [4, 7]. Moreover, mechanisms of acquired resistance to third generation EGFR-TKIs include L718Q or C797S EGFR mutations [8, 9]; in particular, the latter is found in about one third of cancers resistant to osimertinib [10]. The present patient received osimertinib after multiple lines of treatment and obtained a noteworthy PR that lasted over 26 months. Interestingly, clinical outcome was correlated with the number of ex19del ctDNA copies in plasma as it disappeared within a few weeks from the beginning of osimertinib and appeared again and steadily increased thereafter, anticipating tumor progression. The same pattern was seen during chemotherapy, with a decrease in ex19del during disease response and its relapse together with T790M and C797S at PD, suggesting that chemotherapy does not erase cellular clones bearing resistance mutations. Therefore, our data provide evidence that the changes in ex19del in ctDNA are more sensitive than T790M or C797S to monitor osimertinib response and resistance, as T790M appeared 3 months after the increase of activating mutation, and C797S was detectable just before PD. Thus, the efficacy of osimertinib is not simply predicted by T790M; accordingly, previous studies did not show a correlation between T790M levels and benefit of treatment [11–13].

The amount of T790M and C797S in ctDNA at disease progression to gefitinib and osimertinib was markedly lower than ex19del, despite their role in resistance to EGFR-TKIs. This finding is complex to explain due to the primary role of these mutations in driving tumor progression, when it should be expected a similar number of ex19del/T790M/C797S copies in ctDNA. However, a reasonable justification is the presence of multiple metastatic lesions bearing different resistance mutations as a result of clonal evolution.

The present evidence suggests that ex19del reflects tumor biology better than other EGFR molecular alterations, and can be used as a sensitive biomarker to monitor disease outcome. Amplification of EGFR has been documented in cells [14] and in patients resistant to osimertinib [15] and has been shown in one plasma sample of the present patient by NGS; this finding may explain, at least in part, the increase in ex19del copies in ctDNA at each disease progression. Therefore, further analysis should be done to better understand intratumor dynamic clonality as a result of multiple treatments administered to patients. In this respect, the non-invasive molecular analysis of ctDNA may allow a minimally invasive monitoring of disease and provide important information on cancer mutational landscape and emergence of druggable mutations, including c-MET as in the present patient.

In conclusion, this case report provides evidence of the significant activity of osimertinib in a T790M-mutated patient and of the involvement of C797S, EGFR and c-MET amplifications in drug resistance. The changes in ex19del activating mutation in ctDNA effectively predicted disease outcome and was easily detectable due to its larger amount with respect to T790M and C797S. Thus, ex19del measurement is a non-invasive approach to monitor clinical outcome and its increase suggests to perform additional molecular analysis to discover the druggable mutation responsible of drug resistance and adapt treatment strategy.

## Abbreviations

CT: Computed tomography
ctDNA: Circulating tumor DNA
EGFR: Epidermal Growth Factor Receptor
ex19del: Exon 19 deletion of EGFR
NSCLC: Non-small cell lung cancer
PD: Disease progression
PET: Positron emission tomography
PR: Partial response
SD: Stabilization of disease
TKIs: Tyrosine kinase inhibitors
